# Supplementary material for: Interactions between Trypillian farmers and North Pontic forager-pastoralists in Eneolithic central Ukraine
Source: PLoS One. 2023 Jun 14;18(6):e0285449. doi: 10.1371/journal.pone.0285449 (PMC10266615; doi:10.1371/journal.pone.0285449)
Supplement: S3 File — (PDF) [file pone.0285449.s003.pdf]

Interactions between Trypillian farmers and North Pontic forager-pastoralists in Eneolithic central Ukraine

Alexey G. Nikitin, Mykhailo Videiko, Nick Patterson, Virginie Renson, David Reich

S1-S5 Figs

S1 Fig 2
S2 Fig 3
S3 Fig 4
S4 Fig 5
S5 Fig. 6

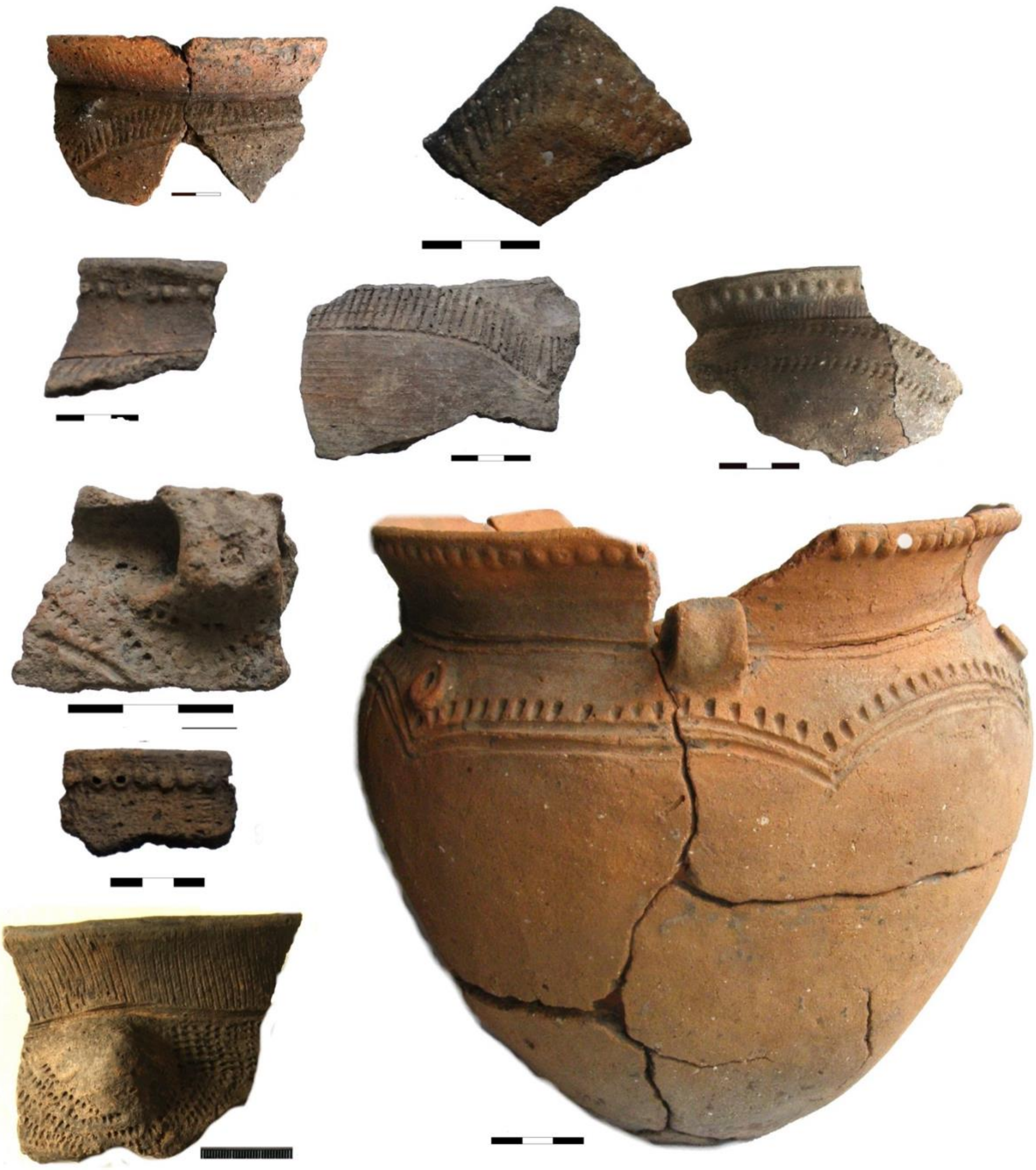

S1 Fig. Cucuteni C pottery from the Trypillian settlement of Kolomiytsiv Yar Tract.

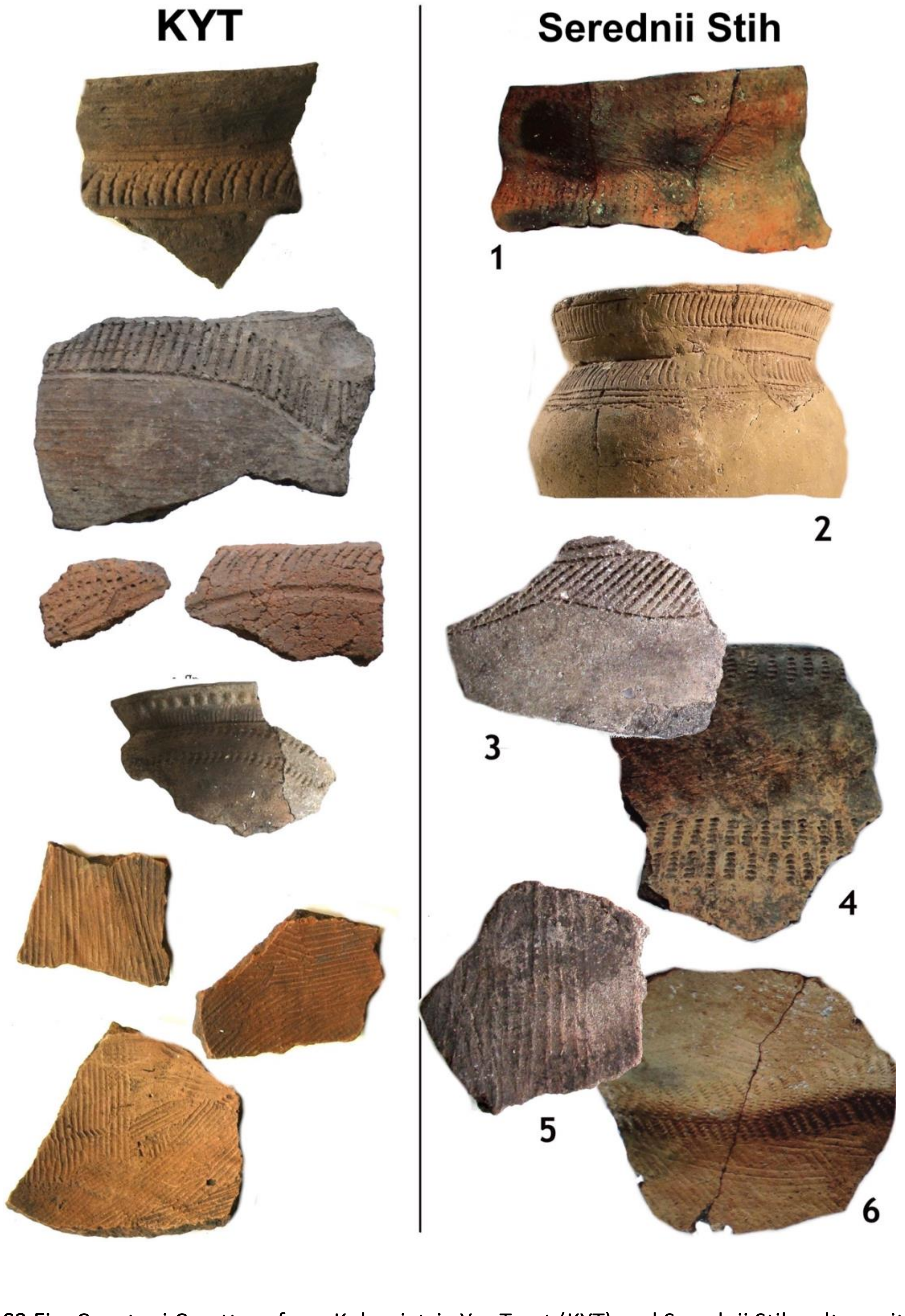

S2 Fig. Cucuteni C pottery from Kolomiytsiv Yar Tract (KYT) and Serednii Stih culture sites. 1,4,6 - Molyukhiv Bugor (after T. Neradenko); 2- Serednii Stih II; 3,5- Stril’cha Skelya.

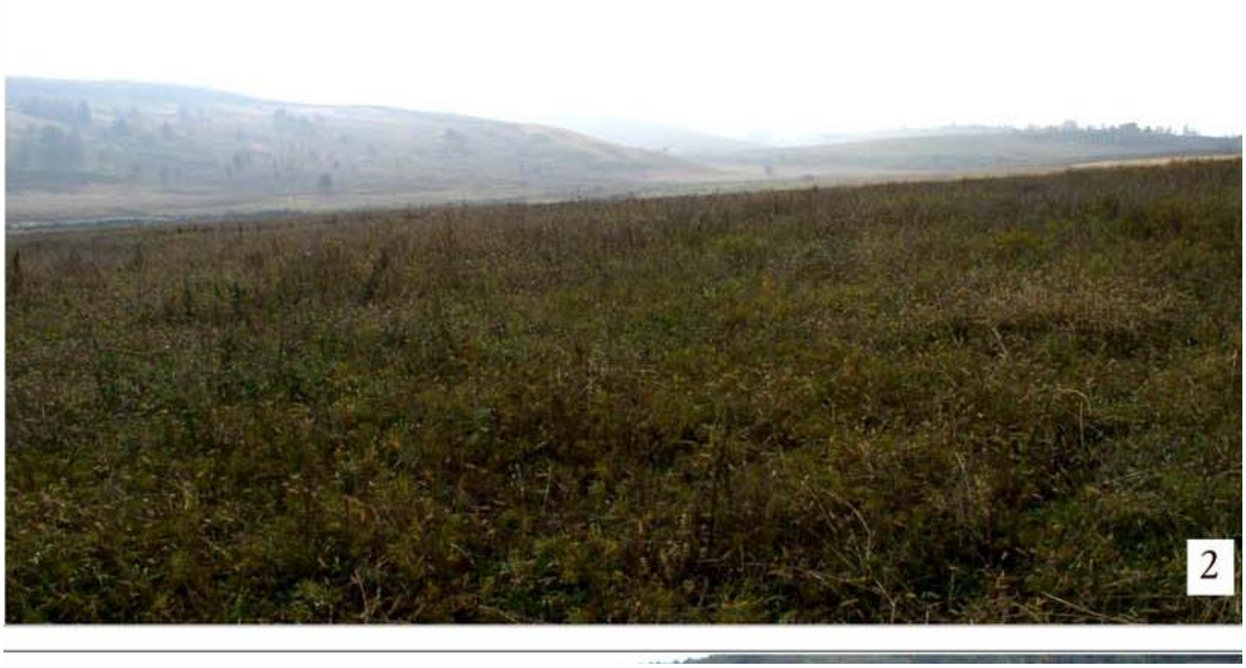

S3 Fig. Kolomiytsiv Yar Tract site location. Photo by M. Y. Videiko, 2006.

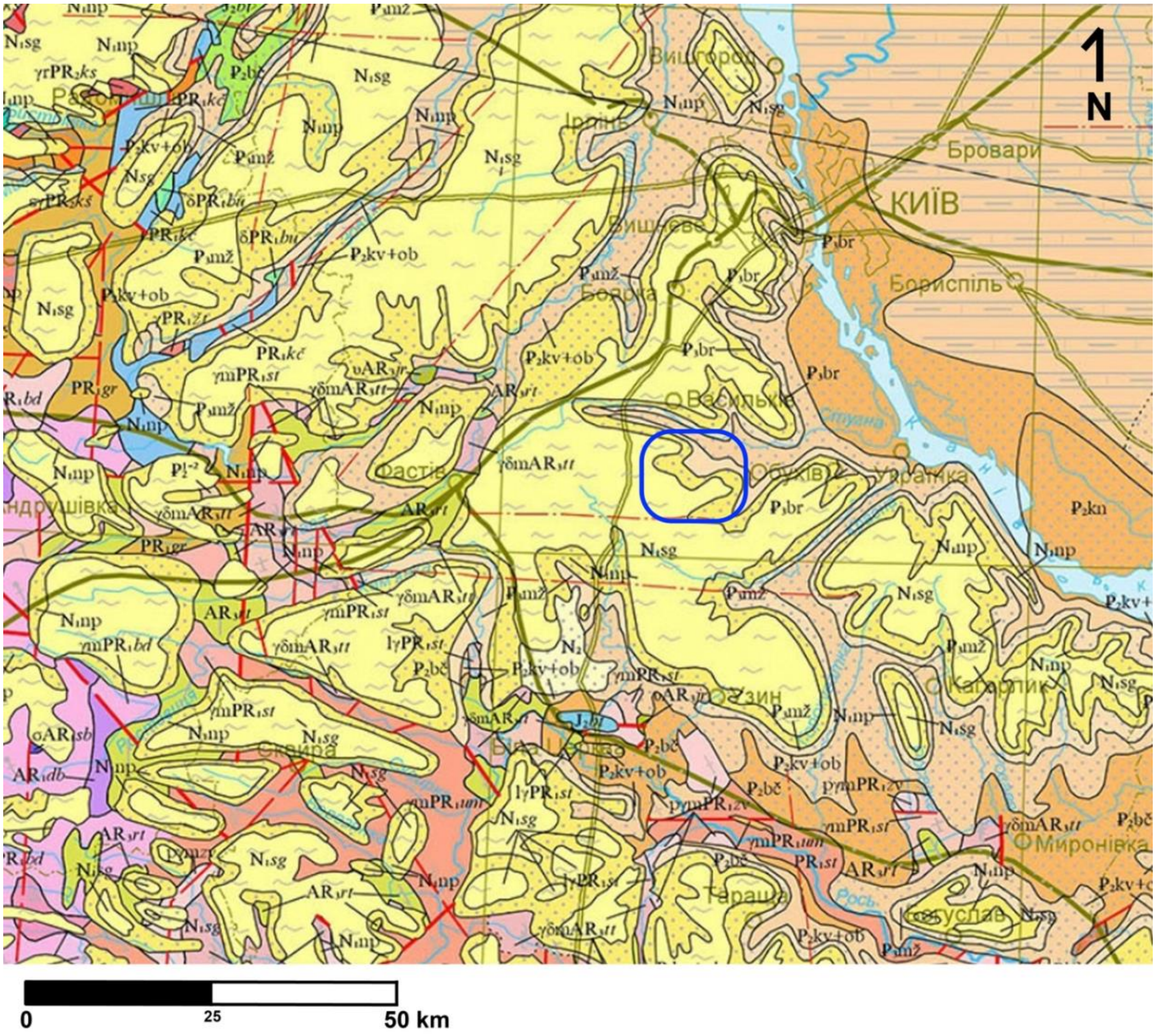

S4 Fig. Regional lithology in the vicinity of the Kolomiytsiv Yar Tract. Image from https://minerals-ua.info/w/mapviewe.php?pr=2 (modified).

The site (outlined in blue) is situated along a small drainage that likely cuts through the following geological units/lithologies:

- N_1np (Miocene) Novopetrivsky Regional Stage – sands, sandstones, clays, including bentonitic and fireproof clays, bands of brown coal
- P_3mz (Oligocene) Mezhyhirsky Regional Stage – glauconitic quartz, argillaceous sands, sometimes – with phosphorite strips
- P_2kv+ob (Eocene) Kyiv and Obukiv Regional Stages – marls, clays, glauconite sands, aleurites, sandstones, including opoka-like sandstones

See https://minerals-ua.info/w/mapviewe.php?pr=2 for detailed legend.

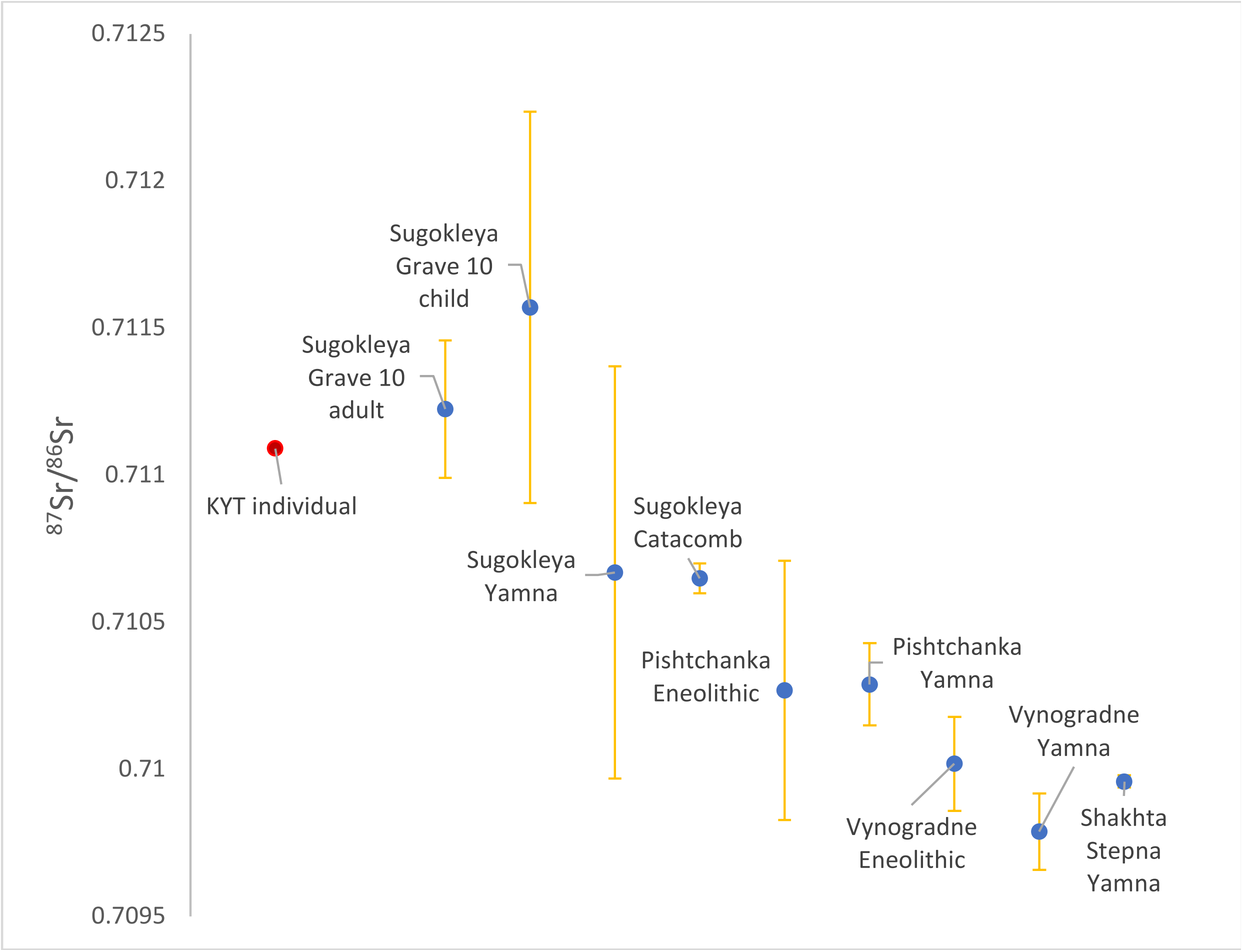

S5 Fig. Strontium isotope ratios from the Kolomiytsiv Yar Tract and Eneolithic and Early Bronze Age (EBA) specimens from the Middle and Lower Dnipro Valley. Geographical coordinates and sources of data are listed in S1 and S4 Tables.
