# Supplementary material for: Interactions between Trypillian farmers and North Pontic forager-pastoralists in Eneolithic central Ukraine
Source: PLoS One. 2023 Jun 14;18(6):e0285449. doi: 10.1371/journal.pone.0285449 (PMC10266615; doi:10.1371/journal.pone.0285449)
Supplement: Attachment — Submitted filename: Response to Reviewers PONE-D-22-34359.docx [file pone.0285449.s004.docx]

April 2, 2023

Re: PLOS ONE Decision: Revision required [PONE-D-22-34359]

Dear Dr. Baca,

Thank you for the opportunity to revise our manuscript. We appreciate the thoughtful comments provided by the reviewers.

We modified the manuscript to address the following two issues raised in your editorial comments and also as the response to Reviewer #2:

1) We clarified the discrepancy in reported numbers of genomic SNPs.
2) We re-wrote the interpretation of isotopic results (please see below for details).

In addition, we modified the manuscript to incorporate the suggestions raised by Reviewer #2 as elaborated below.

Reviewer 2:

“*In the lines 306 to 307, the authors write “Overall, the KYT diet appears comparable to the diet of fisher-forager-pastoralists of the North Pontic steppe, but not to the diet of farmers of Eneolithic Pontic coast and adjacent forest steppe areas (Figure 3)”. In my opinion, this is a contestable statement as the sample plots between all six reference groups and does not cluster with any of them specifically. While the authors also state that “the diet of the KYT individual could also be reflecting a mixture of farming, pastoralist and foraging subsistence practices”, which mitigates the previous assertion, I would advocate for a more cautious conclusion*.”

Response: We re-wrote the paragraph to produce a more cautious assessment of the subsistence practices of the KYT individual and removed the sentence mentioned by the Reviewer.

“*In the segment “Strontium isotope analysis” in the Results and Discussion section (lines 331 to 397), the authors reference several previously published 87Sr/86Sr ratios for Eneolithic and Yamnaya populations from Ukraine. Without knowledge of these publications, it was a difficult for me to follow, thus I wonder if it would be possible to summarise the strontium values in a similar form as it was done for the nitrogen and carbon values in Figure 3*.”

Response: We have added S5 Fig containing a data plot of the strontium ratios of the Eneolithic and Yamna populations from Ukraine.

“*In line 416 the authors write “Whole genome analysis of the KYT specimen produced 76550 Singe Nucleotide Polymorphism “hits” (...)”. I would suggest to rephrase that since it is not clear to me what “whole genome analysis” means in this context. I guess this describes “sequencing of the aDNA libraries after 1240k target enrichment”. In general, since the data is SNP captured and not shotgun sequenced, I would advocate for the use of the term “genome-wide data” instead of “whole genome data”. Also, I cannot not reconstruct why only 76550 SNPs were genotyped, when (according to lines 247 to 248) “a total of 357,145 targeted SNPs on chromosomes [were] covered by at least one sequence”. Additionally, I would like to know the number of SNPs available for PCA. In Table S2, only the 1240k SNPs are reported, yet the PCA was run on Human Origins data, which is only a subset of the 1240k SNP set.*”

Response:

We rephrased the sentence starting on line 416 of the original manuscript to improve clarity. We also added additional details of the Principal Component Analysis in the following sentence.

“*In lines 432 to 436, the authors write “A plausible scenario is that the KYT individual has much of their ancestry from a Proto-Yamna population (Chintalapati et al. 2022), while also being admixed with Serbia/Iron Gates Mesolithic, the latter possibly coming from the Neolithic populations of the Dnipro Valley that have been shown to carry WHG admixture (Mathieson et al. 2018)”. The paper could highly profit from formally testing this hypothesis, e.g. using qpAdm. While PCA and F4-statistics clearly suggest a major proportion of Steppe ancestry in the KYT-SSX individual, qpAdm testing would provide a (citable) percentage and could be furthermore applied to identify the source population that provided the smaller ancestry proportion (which causes the shift away from Yamnaya in the PCA*).”

Response:

Regarding qpAdm, with the data available with this publication we don’t have a good model for this individual’s ancestry. We hope to provide more precise estimates in future publications.

The typos noted by Reviewer #2 have been corrected.

Below please find our response addressing the journal requirements raised in the Decision Letter.

1. We formatted the manuscript according to the style requirements in the provided style templates.
2. A copy of the completed questionnaire on inclusivity in global research is uploaded as Supplementary Information. We also added the “Inclusivity in global research” subsection to the Methods section that includes the suggested sentence.
3. All relevant information about the specimens in the study, including all applicable permits, is provided in the Materials and Methods section. We added a statement about permits and regulations to the text (lines 181-185).
4. Repository information will be provided at manuscript’s acceptance.
5. The map in Fig. 1 was made with Natural Earth, naturalearthdata.com, which is from the list of suggested resources provided in your letter. All versions of Natural Earth raster and vector map data found on their website are in the public domain. Please see <https://www.naturalearthdata.com/about/terms-of-use/> for details.
6. The reference list has been reviewed for completeness and correctness.

We look forward to hearing from you in the near future.

Sincerely,

Alexey G. Nikitin, Ph.D.
